# Association between the ZJU index and gallstone prevalence in a Chinese adult population: a cross-sectional study

**DOI:** 10.3389/fmed.2025.1678281

**Published:** 2025-11-24

**Authors:** Yajie Teng, Daiyi Zhang, Yanjun Chen, Lijuan Qian, Qinhua Xi

**Affiliations:** 1 Department of Gastroenterology, The First People’s Hospital of Kunshan, Suzhou, Jiangsu, China; 2 Health Management Center, The First Affiliated Hospital of Soochow University, Suzhou, Jiangsu, China; 3 Department of Gastroenterology, The First Affiliated Hospital of Soochow University, Suzhou, Jiangsu, China

**Keywords:** ZJU index, gallstones, cross-sectional study, Chinese population, large sample

## Abstract

**Background:**

Gallstone disease is a major health concern, with various metabolic factors implicated in its pathogenesis. The ZJU index, a composite metabolic marker, has been suggested as a potential indicator of metabolic health. However, its association with gallstone disease in the Chinese population remains unclear. This study investigates the relationship between the ZJU index and gallstone prevalence through a cross-sectional analysis.

**Methods:**

We included 55,241 individuals from a Chinese health screening cohort (January–December 2024). Descriptive statistics compared characteristics between individuals with and without gallstones. The ZJU index was calculated for each group, and its association with gallstone presence was assessed using multivariable logistic regression.

**Results:**

After adjusting for confounding variables, a significant positive association was found between the ZJU index and gallstone prevalence (OR = 1.10, 95% CI: 1.04–1.16, *p* < 0.001). Stratified analyses revealed consistent associations in both men (OR = 1.10, 95% CI: 1.03–1.17, *p* = 0.005) and women (OR = 1.13, 95% CI: 1.02–1.25, *p* = 0.022). This positive association was further supported by smooth curve fitting. Formal threshold effect analysis did not identify a statistically significant inflection point (log-likelihood ratio *p* = 0.572), supporting a linear relationship across the range of ZJU index values.

**Conclusion:**

The ZJU index is positively associated with gallstone prevalence in the Chinese population. These findings highlight the potential of the ZJU index as a clinical marker associated with an increased risk for gallstone disease. However, given the cross-sectional nature of this study, causality cannot be inferred, and the observed relationship reflects a correlation rather than a direct cause-and-effect link.

## Introduction

The ZJU (Zhejiang University) index is a composite indicator designed to evaluate overall metabolic health. It is calculated based on body mass index (BMI), fasting plasma glucose (FPG), triglycerides (TG), and the ratio of serum alanine aminotransferase (ALT) to aspartate aminotransferase (AST). Initially proposed in 2015, the ZJU index was developed specifically for the identification of non-alcoholic fatty liver disease (NAFLD) in Chinese populations (1). Compared to single biochemical markers, the ZJU index provides a more integrated assessment of metabolic dysfunction and its associated health risks, thereby offering greater potential for clinical application.

Gallstone disease represents one of the most common disorders of the digestive system. It is estimated that up to 75% of individuals with gallstones are asymptomatic in the early stages. However, as gallstones progress, they may give rise to clinical symptoms such as nausea, epigastric colic, diarrhea, and anorexia. In more severe cases, gallstone obstruction may result in acute and potentially life-threatening complications, including cholangitis, cholecystitis, and biliary pancreatitis (2). Epidemiological studies have shown that the prevalence of gallstone disease in Asian populations is intermediate between that observed in American and African populations (3). Nevertheless, with the increasing adoption of Westernized dietary patterns—characterized by high-calorie, high-carbohydrate, and low-fiber intake—as well as reduced physical activity, the incidence of gallstones has shown a steady upward trend in populations that previously had low baseline prevalence (4, 5).

Gallstones are typically classified into two types: cholesterol stones and pigment stones, each with distinct etiologies. However, the exact biological mechanisms underlying the formation of lithogenic bile remain unclear (6). A growing body of evidence suggests that multiple factors—including age, sex, obesity, and bacterial infection—may contribute to gallstone pathogenesis (7–10). In recent years, increasing attention has been directed toward the link between gallstone disease and metabolic abnormalities, such as metabolic syndrome and metabolic dysfunction-associated fatty liver disease (MAFLD), both of which are considered important contributors to biliary pathology (11–13).

Given that the ZJU index reflects multiple metabolic parameters, it is plausible that it may be associated with the development of gallstone disease. However, current evidence regarding this association in the Chinese population remains scarce. Therefore, this study aims to investigate the cross-sectional association between the ZJU index and gallstone prevalence, to assess whether the ZJU index may serve as a potential marker for gallstone risk stratification in epidemiological contexts.

## Methods

### Study population

**Figure 1:**
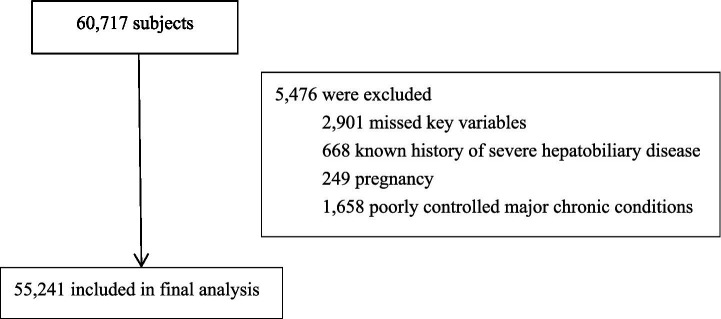
Flow diagram of patient inclusion and exclusion.

This retrospective study analyzed medical records of 60,717 adult individuals who underwent routine health examinations—including blood tests and abdominal ultrasonography—at the First Affiliated Hospital of Soochow University between January and December 2024. All participants completed standardized questionnaires to collect demographic information, medical history, and lifestyle factors, including smoking history (categorized as current smoking, former smoking, or never smoked), alcohol consumption history (categorized as current drinking, former drinking, or never drank), daily exercise (defined as engaging in moderate-intensity aerobic activity for at least 150 min per week or vigorous-intensity activity for at least 75 min per week), as well as histories of diabetes and hypertension. Following this, comprehensive physical assessments were performed. The study was approved by the Ethics Committee of the First Affiliated Hospital of Soochow University. Given the retrospective nature of the research and the use of anonymized secondary data, the requirement for informed consent was waived. A total of 5,476 individuals were excluded based on the following criteria: missing key variables, known history of severe hepatobiliary diseases, pregnancy, or poorly controlled major chronic conditions. A detailed flowchart illustrating the participant selection and exclusion process is presented in Figure 1.

### Laboratory testing and abdominal ultrasound examination

Fasting venous blood samples were collected from the antecubital vein of each participant in the morning after at least 12 h of fasting. Standard enzymatic methods were applied using the LABOSPECT 008 *α* automated biochemical analyzer (HITACHI, Japan) to determine serum levels of aspartate aminotransferase (AST), alanine aminotransferase (ALT), *γ*-glutamyl transferase (GGT), total cholesterol (TC), triglycerides (TG), high-density lipoprotein cholesterol (HDL-C), low-density lipoprotein cholesterol (LDL-C), uric acid (UA), fasting plasma glucose (FPG), and homocysteine (Hcy). The ZJU index was calculated as follows: ZJU index = (ALT/AST) × 3 + BMI (kg/m^2^) + TG (mmol/L) + FPG (mmol/L) + 2 (if female) (1, 14).

Abdominal ultrasound examinations were performed using a ACUSON Redwood color Doppler ultrasound system (Siemens, Germany). All scans were conducted by a senior radiologist who was blinded to laboratory results.

### Statistical analysis

All statistical analyses were conducted using R software (version 3.5.3; R Foundation for Statistical Computing, Vienna, Austria). Continuous variables were expressed as mean ± standard deviation (SD) and compared between groups using the Kruskal–Wallis test. Categorical variables were presented as counts and percentages, with group differences assessed by Fisher’s exact test.

Multivariate logistic regression models were employed to identify potential risk factors associated with gallstone formation, incorporating age, sex, and relevant clinical parameters. Adjusted odds ratios (ORs) and 95% confidence intervals (CIs) were reported. To assess multicollinearity in the fully adjusted models, variance inflation factors (VIFs) were computed, with a threshold of >5 indicating potential concerns. A two-tailed *p*-value <0.05 was considered statistically significant. To evaluate the robustness of the findings, sensitivity analyses were conducted by stratifying the sample by sex. Additionally, generalized additive models (GAMs) were used to explore potential non-linear associations between the ZJU index and gallstone prevalence through smoothed curve fitting. To objectively test for the presence of a non-linear relationship and a potential threshold effect, we employed a two-piecewise linear regression model. The inflection point was estimated using the maximum likelihood method. The superiority of this non-linear model over a simple linear model was formally assessed using the log-likelihood ratio test.

## Results

### Baseline characteristics

Table 1 presents the baseline characteristics of the study participants. A total of 55,241 individuals were included, with 52,635 without gallstones and 2,606 with gallstones. The mean age of participants without gallstones was 43.16 ± 12.38 years, while those with gallstones had a mean age of 51.33 ± 12.74 years, showing a significant difference (*p* < 0.001). The proportion of males was higher in the gallstone group (58.71%) compared to the non-gallstone group (50.23%) (*p* < 0.001). Regarding body measurements, the gallstone group had significantly higher BMI (25.13 ± 3.59 kg/m^2^ vs. 23.96 ± 3.49 kg/m^2^, *p* < 0.001) and waist circumference (84.06 ± 10.63 cm vs. 80.12 ± 10.82 cm, *p* < 0.001). No significant difference in daily exercise prevalence was observed between the groups (*p* = 0.808), but smoking history was more prevalent in the gallstone group (24.48% vs. 18.63%, *p* < 0.001), as was alcohol consumption (15.92% vs. 18.19%, *p* = 0.002). Biochemically, participants with gallstones had elevated levels of alanine aminotransferase (ALT) (25.41 ± 19.51 U/L vs. 23.85 ± 21.30 U/L, *p* < 0.001) and aspartate aminotransferase (AST) (20.66 ± 9.86 U/L vs. 19.97 ± 11.18 U/L, *p* = 0.002). Triglyceride levels were significantly higher in the gallstone group (1.75 ± 1.34 mmol/L vs. 1.54 ± 1.23 mmol/L, *p* < 0.001), while total cholesterol levels did not differ significantly (*p* = 0.727). Homocysteine (11.55 ± 10.81 μmol/L vs. 10.31 ± 5.55 μmol/L, *p* = 0.013) and uric acid (346.24 ± 85.04 μmol/L vs. 335.05 ± 88.11 μmol/L, *p* < 0.001) levels were higher in the gallstone group, as were fasting blood glucose levels (5.36 ± 1.15 mmol/L vs. 5.10 ± 0.98 mmol/L, *p* < 0.001). The prevalence of diabetes (7.37% vs. 3.26%, *p* < 0.001) and hypertension (23.87% vs. 11.51%, *p* < 0.001) was higher in the gallstone group. Additionally, the ZJU index was significantly higher in the gallstone group (36.64 ± 4.94 vs. 35.02 ± 4.88, *p* < 0.001).

**Table 1:** Baseline characteristics of participants.

Characteristic	Without gallstones	With gallstones	*p*-value
*N*	52,635	2,606	
Age	43.16 ± 12.38	51.33 ± 12.74	<0.001
Male	26,437 (50.23%)	1,530 (58.71%)	<0.001
BMI (kg/m^2^)	23.96 ± 3.49	25.13 ± 3.59	<0.001
Waist circumference (cm)	80.12 ± 10.82	84.06 ± 10.63	<0.001
Daily exercise			0.808
Yes	13,159 (25.00%)	646 (24.79%)	
No	39,476 (75.00%)	1,960 (75.21%)	
Smoking			<0.001
Current or ever	9,808 (18.63%)	638 (24.48%)	
Never	42,827 (81.37%)	1,968 (75.52%)	
Drinking			0.002
Current or ever	8,378 (15.92%)	474 (18.19%)	
Never	44,257 (84.08%)	2,132 (81.81%)	
ALT (U/L)	23.85 ± 21.30	25.41 ± 19.51	<0.001
AST (U/L)	19.97 ± 11.18	20.66 ± 9.86	0.002
TC (mmol/L)	4.96 ± 0.91	4.96 ± 0.96	0.727
TG (mmol/L)	1.54 ± 1.23	1.75 ± 1.34	<0.001
HDL-C (mmol/L)	1.32 ± 0.33	1.25 ± 0.31	<0.001
LDL-C (mmol/L)	2.89 ± 0.77	2.91 ± 0.81	0.224
Hcys (umol/L)	10.31 ± 5.55	11.55 ± 10.81	0.013
UA (umol/L)	335.05 ± 88.11	346.24 ± 85.04	<0.001
FBG (mmol/L)	5.10 ± 0.98	5.36 ± 1.15	<0.001
Diabetes			<0.001
Yes	1,715 (3.26%)	192 (7.37%)	
No	50,920 (96.74%)	2,414 (92.63%)	
Hypertension			<0.001
Yes	6,057 (11.51%)	622 (23.87%)	
No	46,578 (88.49%)	1,984 (76.13%)	
ZJU index	35.02 ± 4.88	36.64 ± 4.94	<0.001

BMI, body mass index; ALT, alanine aminotransferase; AST, aspartate aminotransferase; TC, total cholesterol; TG, triglyceride; HDL-C, high-density lipoprotein cholesterol; LDL-C, low-density lipoprotein cholesterol; Hcys, homocysteine; UA, uric acid; FBG, fasting blood glucose; ZJU index, Zhejiang University index.

### Association between ZJU index and gallstone prevalence

Logistic regression analyses demonstrated a significant positive association between the ZJU index and the prevalence of gallstones, as described in Table 2. In the unadjusted model, each 1-point increase in the ZJU index was associated with an approximately 6% increase in the odds of gallstones (OR = 1.06 per 1-point ZJU index increase, 95% CI: 1.05–1.07). This association remained robust after adjusting for gender, age, and waist circumference in Model I (OR = 1.04 per 1-point ZJU index increase, 95% CI: 1.03–1.05, *p* < 0.001). Further adjustment for additional covariates—including hypertension, diabetes, physical activity, smoking, alcohol consumption, uric acid, total cholesterol, and homocysteine—in Model II still revealed a statistically significant association (OR = 1.10 per 1-point ZJU index increase, 95% CI: 1.04–1.16, *p* < 0.001). To enhance the clinical interpretability of the findings, we further analyzed the association per standard deviation (SD) increase in the ZJU index. In the fully adjusted model, each 1-SD increment in the ZJU index was associated with a 59% higher likelihood of gallstone presence (OR = 1.59; 95% CI: 1.22–2.08; *p* = 0.005).

**Table 2:** Logistic regression results for the association of ZJU index with gallstone disease.

With gallstones	Non-adjust		Model I		Model II	
	OR, 95% CI	*p*-value	OR, 95% CI	*p*-value	OR, 95% CI	*p*-value
ZJU index	1.06 (1.05, 1.07)	<0.001	1.04 (1.03, 1.05)	<0.001	1.10 (1.04, 1.16)	<0.001
ZJU index per 1 SD	1.34 (1.29, 1.39)	<0.001	1.22 (1.14, 1.30)	<0.001	1.59 (1.22, 2.08)	<0.001

Model I is adjusted for: gender, age, waist circumference.

Model II is adjusted for: gender, age, waist circumference, hypertension, diabetes, daily exercise, smoking, drinking, UA, TC, Hcys.

Multicollinearity was assessed using the variance inflation factor (VIF), with all covariates in Models showing VIF values below 5, indicating no concerning collinearity (Table 3).

**Table 3:** VIF-based multicollinearity diagnostics for covariates included in adjusted models.

Variable	VIF
Gender	2.9
Age	1.2
Waist circumference	4.1
Hypertension	1.2
Diabetes	1.1
Daily exercise	1.1
Smoking	1.5
Drinking	1.5
UA	1.8
TC	1.1
Hcys	1.1

All values were below the conventional threshold of 5.

### Association between ZJU index and gallstone prevalence in both sexes

Sex-stratified logistic regression analyses revealed a significant positive association between the ZJU index and gallstone prevalence in both men and women, as described in Table 4. In men, each 1-point increase in the ZJU index was associated with a 4% higher odds of gallstones in the unadjusted model (OR = 1.04 per 1-point ZJU index increase, 95% CI: 1.03–1.05, *p* < 0.001). This association remained significant after adjusting for age and waist circumference in Model I (OR = 1.04 per 1-point ZJU index increase, 95% CI: 1.02–1.05, *p* < 0.001). In Model II, which further adjusted for hypertension, diabetes, physical activity, smoking, alcohol consumption, uric acid, total cholesterol, and homocysteine, the association remained statistically significant (OR = 1.10 per 1-point ZJU index increase, 95% CI: 1.03–1.17, *p* = 0.005). Similarly, in women, each 1-point increase in the ZJU index was associated with a 10% higher odds of gallstones in the unadjusted model (OR = 1.10 per 1-point ZJU index increase, 95% CI: 1.08–1.11, *p* < 0.001). After adjusting for age and waist circumference in Model I, this association remained significant (OR = 1.05 per 1-point ZJU index increase, 95% CI: 1.03–1.08, *p* < 0.001). Further adjustment for additional covariates in Model II also revealed a significant association (OR = 1.13 per 1-point ZJU index increase, 95% CI: 1.02–1.25, *p* = 0.022).

**Table 4:** Logistic regression analysis between ZJU with gallstones with subgroup analyses by gender.

With gallstones	Non-adjust		Model I		Model II	
	OR, 95% CI	*p*-value	OR, 95% CI	*p*-value	OR, 95% CI	*p*-value
Male						
ZJU index	1.04 (1.03, 1.05)	<0.001	1.04 (1.02, 1.05)	<0.001	1.10 (1.03, 1.17)	0.005
ZJU index per 1 SD	1.21 (1.15, 1.26)	<0.001	1.20 (1.11, 1.29)	<0.001	1.58 (1.15, 2.18)	0.005
Female						
ZJU index	1.10 (1.08, 1.11)	<0.001	1.05 (1.03, 1.08)	<0.001	1.13 (1.02, 1.25)	0.022
ZJU index per 1 SD	1.56 (1.47, 1.66)	<0.001	1.28 (1.14, 1.44)	<0.001	1.78 (1.09, 2.93)	0.022
Interaction term *p*-value	<0.001		0.346		0.798	

Model I is adjusted for: age, waist circumference.

Model II is adjusted for: age, waist circumference, hypertension, diabetes, daily exercise, smoking, drinking, UA, TC, Hcys.

When examining the ZJU index per standard deviation (SD), in men, each 1-SD increase in the ZJU index was associated with a 21% higher odds of gallstones in the unadjusted model (OR = 1.21, 95% CI: 1.15–1.26, *p* < 0.001). This association remained robust after adjusting for age and waist circumference (OR = 1.20, 95% CI: 1.11–1.29, *p* < 0.001) and was further confirmed after additional adjustments in Model II (OR = 1.58, 95% CI: 1.15–2.18, *p* = 0.005). In women, a 1-SD increase in the ZJU index was associated with a 56% higher odds of gallstones in the unadjusted model (OR = 1.56, 95% CI: 1.47–1.66, *p* < 0.001). After adjustment for age and waist circumference (Model I), the odds ratio decreased slightly to 1.28 (95% CI: 1.14–1.44, *p* < 0.001), and further adjustment for additional confounders in Model II showed an even higher odds ratio of 1.78 (95% CI: 1.09–2.93, *p* = 0.022).

In the formal statistical interaction analysis between sex and the ZJU index, significant results were observed only in the unadjusted model. Specifically, the interaction term between sex and the ZJU index showed a significant association with gallstone prevalence in men and women (*p* < 0.001). However, this interaction was no longer statistically significant in either Model I or Model II (*p* > 0.05 in both cases), suggesting that after adjustment for covariates, the differential effect of the ZJU index on gallstone prevalence between sexes was not significant.

### Non-linear exploration confirms a linear association between ZJU index and gallstone prevalence

**Figure 2:**
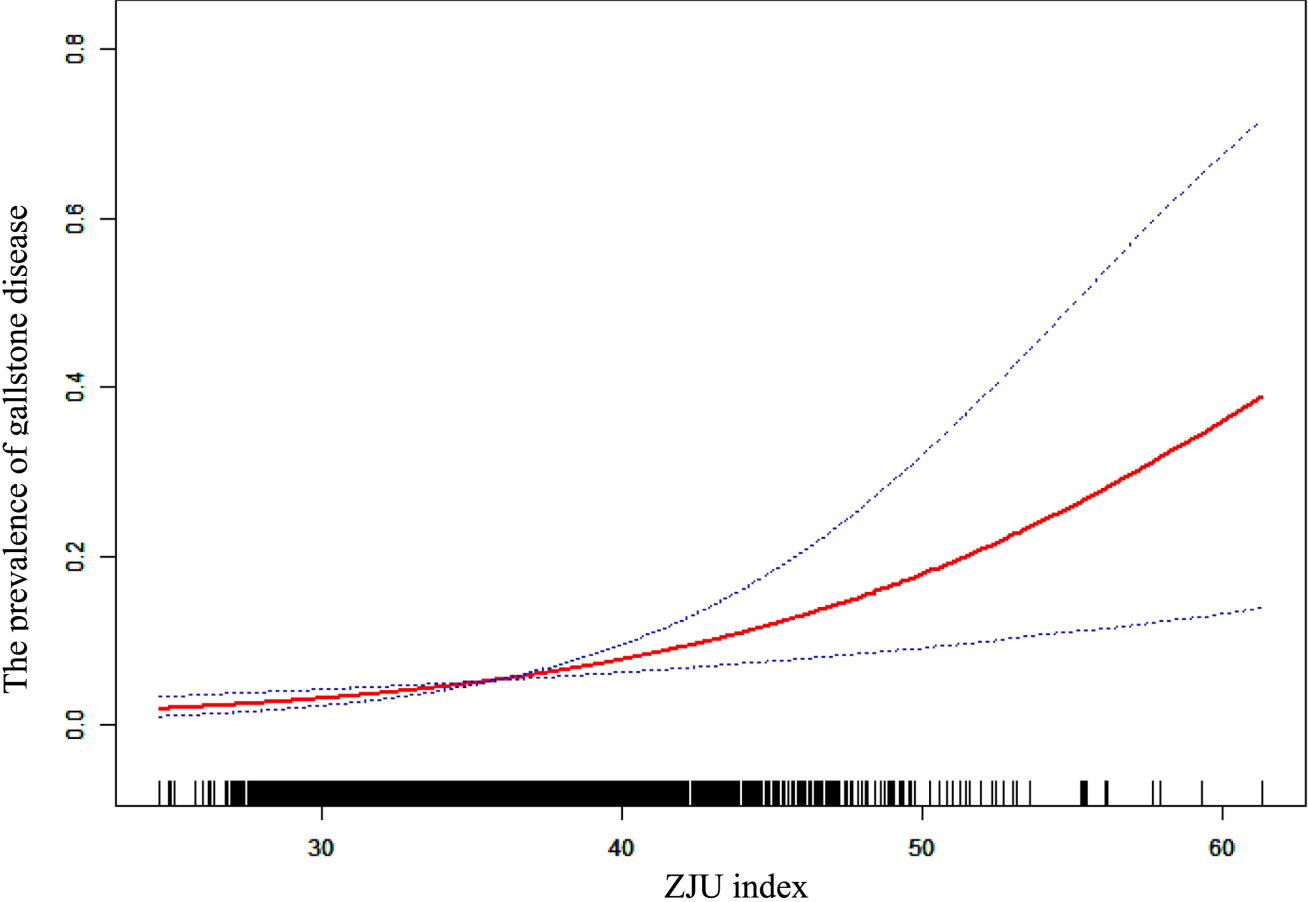
The association between ZJU index and gallstone prevalence. A solid red line represents the smooth curve fit between variables. Blue bands represent the 95% of confidence interval from the fit. The log-likelihood ratio test for non-linearity was not statistically significant (*p* = 0.572), supporting a linear association.

To explore the potential non-linearity in the relationship between the ZJU index and gallstone prevalence, we performed a smooth curve fitting analysis as depicted in Figure 2. The smooth curve suggested a potential non-linear trend, characterized by a shallower slope at lower ZJU index levels which appeared to steepen at higher levels. To further explore the potential threshold effect, a two-piece wise linear regression model was applied, as described in Table 5. We formally tested for the presence of a threshold effect using a two-piecewise linear regression model. The log-likelihood ratio test comparing this model to a simple linear model was not statistically significant (*p* = 0.572). This indicates that the model with a threshold did not provide a statistically superior fit to the data. Therefore, the association between the ZJU index and gallstone prevalence is best characterized by a consistent, linear relationship across the entire range of ZJU index values. The results from the linear model are presented as the primary finding.

**Table 5:** Test for non-linearity in the association between the ZJU index and gallstone occurrence.

Test	*p*-value
Log-likelihood ratio test (two-piecewise vs. linear model)	0.572

Gender, waist circumference, hypertension, diabetes, age, daily exercise, smoking, drinking, UA, TC, Hcys were adjusted. A non-significant *p*-value (*p* > 0.05) indicates that the two-piecewise linear regression model does not provide a better fit to the data than the simple linear model.

## Discussion

Gallstone disease affects approximately 10–20% of the adult population worldwide, representing a major global health concern. More than 20% of individuals with gallstones will develop symptomatic disease—including biliary colic, cholecystitis, or biliary tract infections—typically manifesting in adulthood. Gallstone disease is one of the most economically burdensome gastrointestinal disorders, with substantial direct and indirect healthcare costs (15). Currently, pharmacological prophylaxis is not routinely recommended for the prevention of gallstones, and lifestyle modifications, such as dietary and weight management interventions, remain the primary preventive strategies (16, 17). Therefore, the early identification of high-risk individuals is of paramount importance, both for reducing morbidity and for alleviating the broader socioeconomic burden associated with gallstone disease. Despite increasing awareness, there remains a lack of comprehensive models that characterize the association between integrated metabolic parameters and gallstone prevalence, particularly in Asian populations where disease burden and metabolic profiles may differ from Western cohorts. Thus, our study aims to address this gap by examining the relationship between composite metabolic indices and gallstone occurrence, thereby contributing preliminary evidence that may inform future risk assessment frameworks, although prospective validation is warranted.

In this large-scale cross-sectional study involving over 50,000 Chinese adults, we observed a significant positive association between the ZJU index, a composite metabolic score, and the prevalence of gallstone disease. Multivariable logistic regression analyses indicated that higher ZJU index values were independently associated with increased odds of gallstone formation, even after adjusting for a wide range of demographic, clinical, and biochemical variables. Subgroup analyses revealed that this association was consistent across both sexes. Smooth curve fitting was applied to visually assess the relationship, and consistent with the linear regression results, it demonstrated an overall upward trend in the prevalence of gallstones as the ZJU index increased. These findings indicate that higher ZJU index values are associated with a higher prevalence of gallstone disease in the Chinese population, suggesting its potential utility as a metabolic indicator worthy of further investigation.

The observed association between the ZJU index and gallstone prevalence is biologically plausible, as the ZJU index reflects a constellation of metabolic disturbances, many of which have been individually implicated in the pathogenesis of gallstone disease. The mechanisms underlying this association are likely multifactorial. As an integrated surrogate of metabolic syndrome (MetS), the ZJU index encompasses key components such as obesity, dyslipidemia, and insulin resistance—each of which has been identified as a risk factor for cholelithiasis. Stender et al. (18), using a Mendelian randomization approach, demonstrated a causal relationship between elevated body mass index (BMI) and an increased risk of symptomatic gallstone disease, particularly in women. Smelt (19) further emphasized the strong association between elevated triglyceride (TG) levels and gallstone disease, suggesting potential mechanisms including a close interplay between bile acid (BA) and TG metabolism. Nuclear receptors such as FXR (farnesoid X receptor) and LXR (liver X receptor) play central roles in regulating both BA synthesis and lipid homeostasis. Moreover, individuals with hypertriglyceridemia (HTG) often exhibit impaired gallbladder motility and cholesterol supersaturation in bile—features commonly linked with obesity. Although lipid-lowering therapies such as fibrates and fish oil may improve metabolic profiles and gallbladder function, fibrates have been shown to paradoxically increase the risk of gallstone formation, possibly by enhancing biliary cholesterol saturation and suppressing BA synthesis. This potential adverse effect warrants further mechanistic investigation.

In addition, Chen et al. (20) reported that TG levels may exert a complex, nonlinear effect on gallstone risk. A prospective cohort study revealed a U-shaped association, whereby moderately elevated TG levels were associated with the highest risk of gallstone disease, while risk declined when TG exceeded approximately 2.57 mmol/L. Mendelian randomization analysis further supported elevated TG as an independent causal risk factor for gallstone formation, suggesting TG may influence lithogenesis via alterations in bile composition and gallbladder kinetics. These findings indicate that the effect of TG on gallstone formation is not solely dependent on its absolute concentration but may also be modulated by individual metabolic context and interactions with other lipid parameters. Moreover, Tsai et al. (21), in a large-scale prospective cohort study with 16 years of follow-up, identified 5,771 new cases of cholecystectomy and found that high dietary intake of carbohydrates—particularly those with high glycemic index and glycemic load—was significantly associated with increased risk of gallstones and cholecystectomy in women. This association remained robust after adjusting for confounders, suggesting that such diets may promote gallstone formation through exacerbation of insulin resistance. Chen et al. (22) also demonstrated that gallstone disease is strongly associated with the presence and severity of MetS, with a stepwise increase in gallstone disease prevalence observed with the accumulation of MetS components. Taken together, these findings support the hypothesis that the ZJU index, as a composite marker reflecting multiple metabolic abnormalities, may contribute to gallstone pathogenesis through several interrelated mechanisms including dyslipidemia, obesity, insulin resistance, and impaired gallbladder function. While this underscores the clinical relevance of the ZJU index in reflecting metabolic profiles associated with gallstone presence in population-based settings, prospective validation remains necessary to confirm these findings.

Therefore, the components of the ZJU index—BMI, fasting glucose, triglycerides, and the ALT/AST ratio—are closely linked to insulin resistance, hepatic steatosis, and disrupted lipid metabolism. These factors are associated with bile cholesterol supersaturation, gallbladder hypomotility, and an increased risk of cholesterol crystal formation, all of which create a biological environment that promotes gallstone formation. Although the smooth curve visually suggested a potential non-linear trend, the formal threshold effect analysis did not support it. Therefore, the association is best interpreted as a linear, gradual increase in gallstone risk across the entire range of ZJU index values. Given the cross-sectional association observed in this study, the ZJU index may serve as a simple and accessible surrogate marker reflecting metabolic profiles associated clinical utility of the ZJU index in understanding metabolic factors linked to gallstones. Further longitudinal studies are warranted to examine the association between the ZJU index and the future development of gallstones. Our findings could contribute to developing risk stratification models for gallstones. For example, individuals with a high ZJU index may benefit from ultrasound screening during routine health check-ups, given the index’s ease of use. Those identified with asymptomatic stones or at high risk could then receive counseling on lifestyle changes, such as weight management and dietary adjustments, to prevent progression or complications.

Our findings are generally consistent with those of the U.S. NHANES 2017–2020 study, which also reported a positive association between the ZJU index and gallstone prevalence (23). However, it is worth noting that the NHANES study focused on a multi-ethnic Western population, which may limit the direct applicability of its results to Chinese individuals, for whom the ZJU index was originally developed. By concentrating on a large Chinese cohort, our study provides population-specific evidence that further supports the potential of the ZJU index as a useful tool for assessing gallstone risk. Although both studies show a similar trend, the observed association between the ZJU index and gallstone prevalence may differ due to ethnic, lifestyle, and genetic factors that influence metabolic profiles and gallstone formation. For instance, dietary habits, the prevalence of metabolic disorders, and genetic predispositions within the Chinese population could result in different patterns of association compared to those observed in Western populations. Additionally, differences in healthcare access and preventive measures between the populations might also account for variations in the strength of this association. Our findings contribute to a deeper understanding of the role of metabolic markers in gallstone prevalence and highlight the potential value of incorporating the ZJU index into routine health assessments in China. Given its simplicity and relevance to the Chinese population, the ZJU index may serve as an effective tool for identifying individuals at higher risk for gallstones. This could facilitate early identification and inform potential interventions targeting individuals with elevated risk markers, in alignment with the specific needs of the Chinese population.

This study has several strengths. The large sample size enhances statistical power, and standardized data collection ensures high reliability. The use of multivariable models and nonlinear analysis allows for a more accurate assessment of the ZJU index’s association with gallstones. Stratified analysis further supports the robustness of the findings. However, several limitations must be acknowledged. Although we adjusted for key covariates such as age, sex, waist circumference, hypertension, diabetes, physical activity, smoking, alcohol use, uric acid, cholesterol, and homocysteine, other important risk factors were not included. These factors, such as diet, genetic predispositions, and family history, could confound the observed association. Specifically, dietary factors like high-fat or high-carbohydrate diets are known to affect both metabolic health and gallstone formation (24). Additionally, we did not distinguish between cholesterol and pigment stones, which have different etiologies. Cholesterol stones are primarily influenced by metabolic factors such as obesity and dyslipidemia (25), while pigment stones are associated with conditions like hemolysis and infection (3). The lack of gallstone subtype data may lead to misclassification and could obscure the true association. Furthermore, the study participants were recruited from a single center, which may limit the generalizability of the findings. Future studies should employ longitudinal designs to better understand causal relationships. Investigating underlying mechanisms and validating these findings in broader, more diverse populations may contribute to understanding the potential role of the ZJU index in clinical assessments related to gallstone risk.

## Conclusion

In conclusion, this study demonstrates a significant association between the ZJU index and the prevalence of gallstones in a general adult population. As an integrated marker reflecting metabolic status, the ZJU index may serve as a practical and cost-effective tool for individuals at elevated risk of gallstone disease. However, due to the cross-sectional nature of the study, causal relationships cannot be established. Prospective cohort studies and mechanistic investigations are warranted to validate these findings and elucidate the potential pathophysiological pathways linking metabolic dysfunction and gallstone formation.

## Data Availability

The raw data supporting the conclusions of this article will be made available by the authors, without undue reservation.
